# Psychological morbidity and associated factors among perinatal patients referred for psychiatry assessments at a tertiary care centre in Sri Lanka

**DOI:** 10.1192/bjo.2021.766

**Published:** 2021-06-18

**Authors:** Saumya Madhri Senanayake, Iresha Perera, Janith Galhenage, Raveen Hanwella

**Affiliations:** Professorial Psychiatry Unit, National Hospital of Sri Lanka

## Abstract

**Aims:**

Our objective was to study the psychological morbidity and associated risk factors among antenatal and postnatal patients referred for the psychiatric assessment at University Psychiatry Unit of National Hospital of Sri Lanka.

**Method:**

All the Clinic records of perinatal referrals from 1st January 2019 to 31st December 2019 were assessed. Sociodemographic details, delivery details, health of the newborn, past and present psychiatry illness related details were obtained using a questionnaire. Data were analysed using SPSS.

**Result:**

Total of 161 perinatal referrals were studied. Mean age of the mothers were 28.7 years (SD = 6.60). About 18 (11.8%) were not legally married, partner passed away or estranged. Above Ordinary level education was having 34.5% of participants. Majority were postnatal mothers (61.5%). Some mothers (32.3%) have reported the pregnancy was unexpected whilst 20(32.3%) and 49(30.8%) have experienced delivery complications and neonatal illnesses respectively. Past mental illnesses were found among 31(20.7%) of mothers. Out of whole perinatal referrals maternity blues (28.9%) was the commonest current psychiatry diagnosis. Among antenatal mothers, adjustment disorder (28.8%) and depressive disorder (17.3%) were the commonest. Schizophrenia, Schizophreniform disorder and bipolar illness were found among 8(5%), 6(3.7%) and 3(1.9%) mothers respectively. Major psychoactive substance use disorder was found among 4 (2.5%) mothers. Presence of pregnancy related complications were significantly associated with postpartum metal illnesses(p = 0.008).

**Conclusion:**

Commonest perinatal mental illness was the maternity blues. Depressive disorder was the commonest major mental illness and neonatal complications were associated with psychological morbidity in postnatal mothers.

